# A phlorotannin constituent of *Ecklonia cava* alleviates postprandial hyperglycemia in diabetic mice

**DOI:** 10.1080/13880209.2017.1291693

**Published:** 2017-02-20

**Authors:** Hyun-Ah Lee, Ji-Hyeok Lee, Ji-Sook Han

**Affiliations:** a Department of Food Science and Nutrition, Pusan National University, Busan, Republic of Korea;; b Korea Mouse Metabolic Phenotyping Center, Jeju, Republic of Korea;; c Department of Food Science and Nutrition and Research, Pusan National University, Busan, Republic of Korea;; d Institute of Ecology for the Elderly, Pusan National University, Busan, Republic of Korea

**Keywords:** α-Glucosidase, α-amylase, streptozotocin

## Abstract

**Context**: 2,7″-Phloroglucinol-6,6′-bieckol is a type of phlorotannin isolated from brown algae, *Ecklonia cava* Kjellman (Phaeophyceae; Laminareaceae). 2,7″-Phloroglucinol-6,6′-bieckol mediates antioxidant activities. However, there has been no research on improving postprandial hyperglycaemia using 2,7″-phloroglucinol-6,6′-bieckol.

**Objective:** This study investigated the inhibitory effects of 2,7″-phloroglucinol-6,6′-bieckol on activities of α-glucosidase and α-amylase as well as its alleviating effect on postprandial hyperglycaemia in streptozotocin-induced diabetic mice.

**Materials and methods:** α-Glucosidase and α-amylase inhibitory assays were carried out. The effect of 2,7″-phloroglucinol-6,6′-bieckol on hyperglycaemia after a meal was measured by postprandial blood glucose in streptozotocin-induced diabetic and normal mice. The mice were treated orally with soluble starch (2 g/kg BW) alone (control) or with 2,7″-phloroglucinol-6,6′-bieckol (10 mg/kg bw) or acarbose (10 mg/kg BW) dissolved in 0.2 mL water. Blood samples were taken from tail veins at 0, 30, 60, and 120 min and blood glucose was measured by a glucometer.

**Results:** 2,7″-Phloroglucinol-6,6′-bieckol showed higher inhibitory activities than acarbose, a positive control against α-glucosidase and α-amylase. The IC_50_ values of 2,7″-phloroglucinol-6,6′-bieckol against α-glucosidase and α-amylase were 23.35 and 6.94 μM, respectively, which was found more effective than observed with acarbose (α-glucosidase IC_50_ of 130.04 μM; α-amylase IC_50_ of 165.12 μM). In normal mice, 2,7″-phloroglucinol-6,6′-bieckol significantly suppressed the postprandial hyperglycaemia caused by starch. The 2,7″-phloroglucinol-6,6′-bieckol administration group (2349.3 mmol·min/L) had a lower area under the curve (AUC) glucose response than the control group (2690.83 mmol·min/L) in diabetic mice.

**Discussion and conclusion:** 2,7″-Phloroglucinol-6,6′-bieckol might be used as an inhibitor of α-glucosidase and α-amylase as well as to delay absorption of dietary carbohydrates.

## Introduction

Diabetes mellitus is a progressive metabolic disorder characterized by high blood glucose levels (Sheetz 2002). Especially, a postprandial hyperglycaemia state is an important contributing factor to the development of type 2 diabetes mellitus as well as related complications, including atherosclerosis, diabetic nephours opathy and neuropathy. Therefore, control of hyperglycaemia is the most important factor for reducing risk of diabetic complications and is a major goal of diabetes treatment (Bonora & Muggeo 2001; Fujita et al. 2001). The most effective way to control postprandial blood glucose levels is medication in combination with dietary restriction and an exercise program (Yki-Jarvinen 1990). Available medications for diabetes include insulin and various oral hypoglycaemic agents such as sulfonylureas, biguanides, α-glucosidase inhibitors, etc. These drugs are used as monotherapies or in combination to achieve improved glycaemic control. However, each of the above oral antidiabetic agents is associated with a number of serious adverse effects. Hence, antidiabetic drugs have been recently screened and developed from natural sources with minimal side effects (Sels et al. 1999; Standl et al. 1999).

*Ecklonia cava* Kjellman (Phaeophyceae; Laminareaceae) is an edible marine brown alga species found in the oceans of Korea and Japan. *Ecklonia cava* has received attention recently due to its various biological activities, including radical scavenging, antiproliferative, antiallergic, antidiabetic and protease inhibitory effects (Ahn et al. 2004; Kang et al. 2005, 2010, 2013; Kim et al. 2008b; Park et al. 2015). These effects are attributed to several compounds such as xanthophyll pigment, fucoxathin, phlorotannins and fucoidans. Especially, *Ecklonia cava* contains an abundance of biological polyphenolic compounds, referred to as phlorotannins. Phlorotannins are reported to possess antioxidant and anti-inflammatory activities but also metalloproteinase inhibitory activities (Li et al. 2009; Wijesekara et al. 2010).

**Figure 1.**
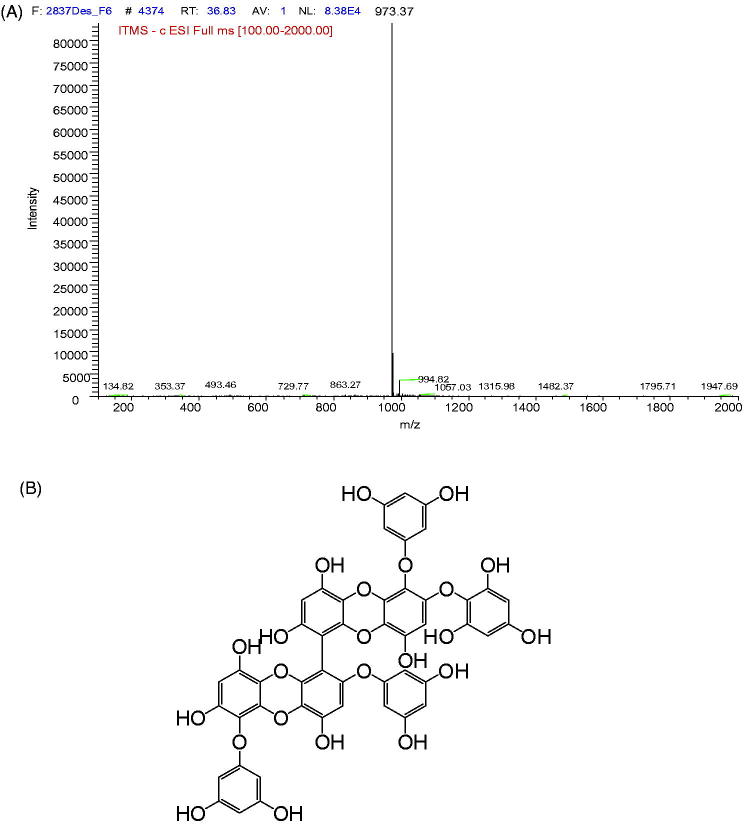
MS spectra and chemical structure of active compound isolated from *E. cava*. MS spectra and chemical structure of the active compound isolated from *E. cava.* The spectra were generated in negative ionization mode (A). Chemical structure and HMBC correlations of 2,7″-phloroglucinol-6,6′-bieckol isolated from *E. cava.* (B).

2,7″-Phloroglucinol-6,6′-bieckol is a type of phlorotannin isolated from brown algae, *Ecklonia cava*. In mass spectrometry (MS), 2,7″-phloroglucinol-6,6′-bieckol showed a M + of 974 *m/z*, corresponding to the molecular formula of C_48_H_30_O_23_ (Figure 1). Previous studies revealed that 2,7″-phloroglucinol-6,6′-bieckol mediates antioxidant activities (Kang et al. 2012; Yotsu-Yamashita et al. 2013). However, there has been no research on improving postprandial hyperglycaemia using 2,7″-phloroglucinol-6,6′-bieckol. Thus, in the present study, we investigated the inhibitory effects of 2,7″-phloroglucinol-6,6′-bieckol isolated from *Ecklonia cava* on α-glucosidase and α-amylase activities as well as its alleviating effect on postprandial hyperglycaemia in streptozotocin (STZ)-induced diabetic mice.

## Materials and methods

### Materials

Brown alga, *Ecklonia cava*, was collected along the coast of Jeju Island, Korea, between February and May of 2012. Verification of vouchers or living alga was performed by department of Faculty of Marine Biomedical Sciences of Jeju National University. Samples were washed three times with water to remove any attached salt, epiphytes, and sand, then rinsed carefully with fresh distilled water, and stored in a medical refrigerator at −20 °C. Thereafter, frozen samples were lyophilized and homogenized using a grinder prior to extraction.

### Extraction and isolation

Dried *Ecklonia cava* powder (500 g) was extracted using 5 L of 80% aqueous methanol three times at room temperature. The liquid layer was obtained via filtration, and the filtrate was concentrated using an evaporator under reduced pressure. The extract was suspended in H_2_O, and the aqueous layer was partitioned with ethyl acetate (EtOAc). The EtOAc extract (45.65 g) was mixed with celite, and the resulting mixture was then dried and packed into a glass column and subsequently eluted in the following order: hexane, dichloromethane, diethyl ether, and butanol. The diethyl ether fraction was subjected to silica column chromatography using a CHCl_3_/MeOH gradient system (2:1 to 100% methanol) to obtain eight sub-fractions (*E. cava*-diethyl ether fraction, ECE). The active compound from ECE7 was isolated via RP-HPLC and acquired as an amorphous, brown powder with a molecular weight of 974 *m/z*, as determined by mass spectrometry (Figure 1(A)). The active compound was structurally identified as 2,7″-phloroglucinol-6,6′-bieckol via spectral analysis.

### 2,7″-Phloroglucinol-6,6′-bieckol

2,7″-Phloroglucinol-6,6′-bieckol: amorphous powder, ^1^H NMR (400 MHz, methanol-*d*4) d 5.57 (1 H,s), 5.89 (1 H,s), 5.74 (1 H,m), 5.84 (1 H,m), 5.74 (1 H,m), 6.25 (1 H,s), 6.14 (1 H,s), 5.84 (1 H,m), 5.89 (1 H,m), 5.84 (1 H,m), 6.52 (1 H,s), 6.14 (1 H,m), 6.44 (1 H,m), 6.77 (1 H,s), 6.72 (1 H,s), 8.93 (1 H,s), 8.93 (1 H,s), 9.19 (1 H,s), 9.19 (1 H,s), 9.19 (1 H,s), 9.04 (1 H,s), 8.26 (1 H,s), 9.94 (1 H,s), 8.59 (1 H,s), 9.88 (1 H,s), 9.86 (1 H,s), 9.25 (1 H,s), 9.75 (1 H,s), 9.21 (1 H,s); ^13^C NMR (100 MHz, methanol-*d*6) d 127.6, 143.0, 93.0, 137.1, 125.6, 147.2, 106.5, 152.2, 95.5, 152.4, 127.6, 137.1, 162.0, 98.7, 160.3, 95.5, 160.3, 98.8, 124.3, 147.2, 94.5, 144.1, 124.3, 147.2, 110.0, 144.1, 101.5, 151.8, 137.2, 144.1, 159.7, 96.7, 157.1, 95.5, 157.1, 96.7, 159.8, 97.8, 159.3, 95.2, 159.2, 97.9, 122.5, 153.9, 99.8, 156.8, 99.9, 152.8(d); ESI-MS:[M-H]^-^ at *m/z* 973.37.

### Cell viability

Cell viability was assessed using a modified 3-(4,5-dimethylthiazol-2yl)-2,5-diphenyl-2H-tetrazolium bromide (MTT) assay. Briefly, cells (2 × 10^4^ cells/well) were seeded in a 96 well plate and treated 2,7″-phloroglucinol-6,6′-bieckol. Following treatment, 100 μL of MTT solution (5 mg/mL in phosphate buffered saline) was added to each well and further incubated for 4 h at 37 °C. Subsequently, 100 μL of dimethyl sulfoxide (DMSO) was added to each well to dissolve any deposited formazan. The optical density (OD) of each well was measured at 540 nm with a microplate reader (Bio-Rad Laboratories Inc., Hercules, CA).

### Inhibition assay for α-glucosidase activity *in vitro*

The α-glucosidase inhibitory assay was carried out by the chromogenic method developed by Watanabe et al. (1997) using a readily available yeast enzyme. Briefly, yeast α-glucosidase (0.7 U, Sigma, St. Louis, MO) was dissolved in 100 mM phosphate buffer (pH 7.0) containing 2 g/L of bovine serum albumin and 0.2 g/L of NaN_3_ and used as an enzyme solution. *p*-Nitrophenyl-α-d-glucopyranoside (5 mM) in the same buffer (pH 7.0) was used as a substrate solution. Enzyme solution (50 μL) and 10 μL of sample dissolved in dimethylsulfoxide at a concentration of 5 mg/mL were mixed in a well, and absorbance at 405 nm was measured using a microplate reader. After incubation for 5 min, substrate solution (50 μL) was added and incubated for another 5 min at room temperature. The increase in absorbance from zero time was measured. Inhibitory activity was expressed as 100 minus the relative absorbance difference (%) of the test compounds compared to the absorbance change of the control where the test solution is replaced by carrier solvent. Measurements were performed in triplicate, and IC_50_ value, i.e., concentration of extracts resulting in 50% inhibition of maximal activity, was determined.

### Inhibition assay for α-amylase activity *in vitro*

The α-amylase inhibitory activity was assayed in the same way (Watanabe et al. 1997) as described for the α-glucosidase inhibitory assay, except that porcine pancreatic amylase (100 U, Sigma, St. Louis, MO) and blocked. *p*-Nitrophenyl-α-d-glucopyranoside (Sigma, St Louis, MO, USA) were used as enzyme and substrate, respectively.

### Experimental animals

Four-week-old male mice (ICR, Orient, Inc., Seoul, Korea) were kept under a 12 h light/dark cycle at room temperature. The animals were provided pelleted food every day, whereas tap water was provided *ad libitum*. After an adjustment period of 2 weeks, diabetes was induced in the fasted (18 h) animals by intraperitoneal injection of STZ (60 mg/kg) freshly dissolved in citrate buffer (0.1 M, pH 4.5). Although STZ-induced diabetic mice were an animal model of type 1 diabetes, it was generally used in the study on the effect of short-term intake such as alleviating effect of postprandial hyperglycaemia. After 7 days, tail bleeds were performed and animals with a blood glucose concentration above 250 mg/dL (14 mM) were considered to be diabetic.

### Measurement of blood glucose level

Both normal and STZ-induced diabetic mice fasted overnight were randomly divided into four groups. Fasted animals were deprived of food for at least 12 h but allowed free access to water. After overnight fasting, mice were orally administered either soluble starch (2 g/kg body weight) alone (control) or starch with 2,7″-phloroglucinol-6,6′-bieckol (10 mg/kg body weight). Blood samples were taken from tail veins at 0, 30, 60, and 120 min. Blood glucose was measured using a glucometer (Roche Diagnostics GmbH, Germany). Areas under the curve (AUC) were calculated using the trapezoidal rule (Kim 2004). All procedures were approved by the animal ethics committee of our university.

### Data statistical analysis

Data were represented as mean ± SD. Statistical analysis was performed using SAS software (SAS Institute, Inc., Cary, NC). Student’s *t*-test was used for comparisons between control and sample groups. Values were evaluated by one-way analysis of variance (ANOVA), followed by *post hoc* Duncan's multiple range tests.

## Results

### Cell viability

**Figure 2.**
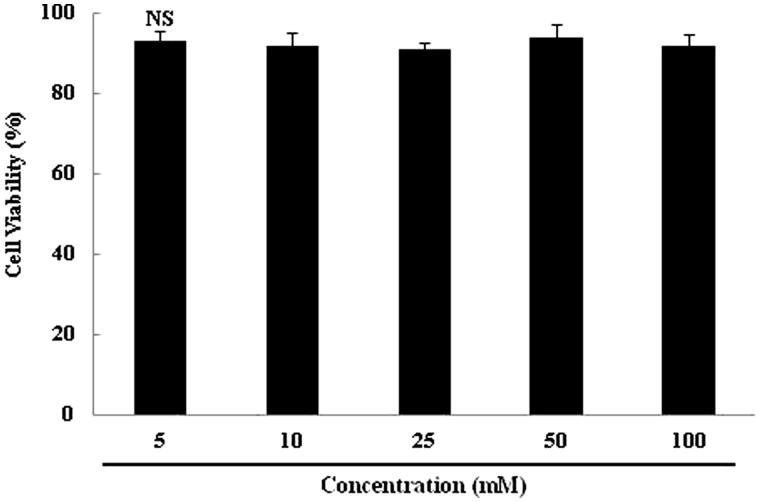
Effect of 2,7″-phloroglucinol-6,6′-bieckol on cytotoxicity in 3T3-L1 cells. Cells in 96-well plates (2 × 10^4^ cells/well) were incubated with and without indicated concentrations of 2,7″-phloroglucinol-6,6′-bieckol for 20 h. Each value is expressed as mean ± SD.

3T3-L1 cell viability in the presence of oyster shell extract is shown in Figure 2. These data indicate that 2,7″-phloroglucinol-6,6′-bieckol does not affect the viability of 3T3-L1 cells at the concentrations of 10–100 μg/mL.

### Inhibitory effects of 2,7″-phloroglucinol-6,6′-bieckol on *α*-glucosidase and *α*-amylase *in vitro*

**Figure 3.**
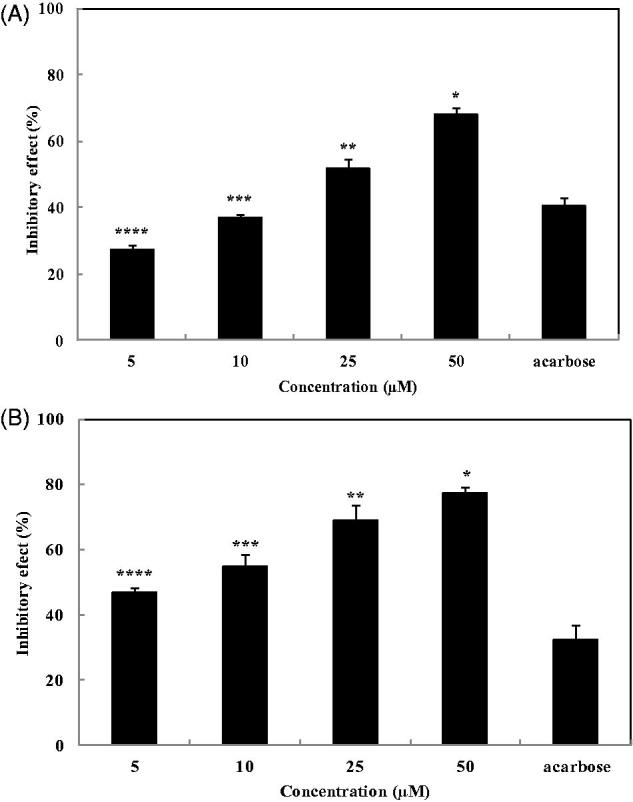
Inhibitory effects of 2,7″-phloroglucinol-6,6′-bieckol against α-glucosidase (A) and α-amylase (B). Acarbose was used as a positive control. Each value is expressed as the mean ± SD of triplicate experiments. Values with different symbols (*,**,***,****) are significantly different at *p* < 0.05 in Duncan’s multiple range tests. The final concentration of acarbose is 100 μM.

The α-glucosidase inhibitory effect of 2,7″-phloroglucinol-6,6′-bieckol was determined (Figure 3(A)). 2,7″-Phloroglucinol-6,6′-bieckol inhibited α-glucosidase activity in a dose-dependent manner by 27.17, 37.09, 51.59, and 68.06% at concentrations of 5, 10, 25, and 50 μM, respectively. The α-glucosidase inhibitory activity of 2,7″-phloroglucinol-6,6′-bieckol at a concentration of 10 μM was similar to that of acarbose at a concentration of 100 μM. The inhibitory effect of 2,7″-phloroglucinol-6,6′-bieckol extract against α-amylase activity is shown in Figure 3(B). 2,7″-Phloroglucinol-6,6′-bieckol inhibited α-amylase activity by 46.96, 54.78, 69.13, and 77.39% at concentrations of 5, 10, 25, and 50 μM. At a concentration of 5 μM, α-amylase inhibitory activity of 2,7″-phloroglucinol-6,6′-bieckol (46.96%) was higher than that of acarbose (32.57% at concentration of 100 μM). Corroborating these results, 2,7″-phloroglucinol-6,6′-bieckol exerted strong inhibitory activity not only against α-glucosidase (IC_50_ of 23.35 μM) but also α-amylase (IC_50_ of 6.94 μM) (Table 1).

**Table 1.** IC_50_ values of inhibitory effects of 2,7″-phloroglucinol-6,6′-bieckol on α-glucosidase and α-amylase.

	IC_50_ (μM)^a^^)^	
Sample	α-Glucosidase	α-Amylase
2,7″-Phloroglucinol-6,6′-bieckol	23.35 ± 4.38^*b)^	6.94 ± 1.07*
Acarbose	130.04 ± 8.42	165.12 ± 6.19

a The IC_50_ value is the concentration of sample required for 50% inhibition. Each value is expressed as the mean ± S.D. in triplicate experiments.

b Symbol (*) is significantly different at *p* < 0.05 in Duncan’s multiple range tests.

### Effect of 2,7″-phloroglucinol-6,6′-bieckol on blood glucose level *in vivo*

**Figure 4.**
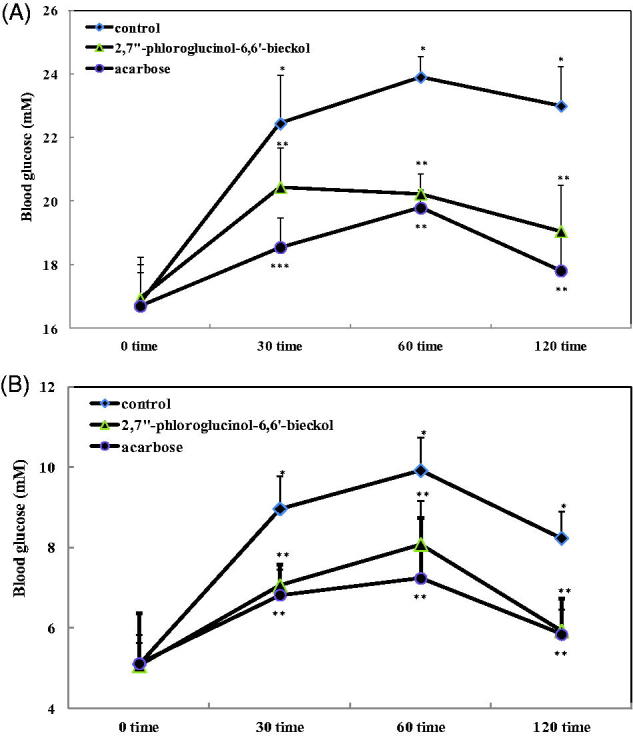
Blood glucose levels after administration of 2,7″-phloroglucinol-6,6′-bieckol in streptozotocin-induced diabetic mice (A) and normal mice (B). 2,7″-Phloroglucinol-6,6′-bieckol (10 mg/kg), acarbose (10 mg/kg), and control (distilled water) were orally co-administered starch (2 g/kg). Each value is expressed as the mean ± S.D. of seven mice (*n* = 42). Values with different symbols (*, **) are significantly different at *p* < 0.05 in Duncan’s multiple range tests.

To evaluate the effect of 2,7″-phloroglucinol-6,6′-bieckol on hyperglycaemia after a meal, postprandial blood glucose was measured in STZ-induced diabetic and normal mice. In diabetic mice, blood glucose levels of the control group increased to 22.44, 23.91 and 23.00 mM at 30, 60 and 120 min, respectively (Figure 4(A)). However, the 2,7″-phloroglucinol-6,6′-bieckol group showed significantly reduced blood glucose levels (20.44, 20.23, and 19.06 mM at 30, 60 and 120 min, respectively) as well as a smaller AUC of the glucose response curve (2349.31 ± 129.17 mmol·min/L) compared to the control group (2690.83 ± 152.88 mmol·min/L, *p <* 0.05, Table 2). For blood glucose level, there were no significant differences between the 2,7″-phloroglucinol-6,6′-bieckol and acarbose groups at 60 and 120 min. Further, 2,7″-phloroglucinol-6,6′-bieckol and acarbose (oral hypoglycemic agent used as positive control) groups showed similar AUC values. In control normal mice, blood glucose level increased to 10.86 mM at 60 min after starch load (Figure 4(B)). Normal mice administered starch with 2,7″-phloroglucinol-6,6′-bieckol showed significantly decreased blood glucose levels (7.06, 8.08, and 5.93 mM at 30, 60, and 120 min, respectively) and AUC values (828.78 ± 85.30 mmol/min/L) compared to the control group (1038.53 ± 91.75 mmol/min/L). The acarbose and 2,7″-phloroglucinol-6,6′-bieckol groups showed similar patterns for both blood glucose levels and AUC values.

**Table 2.** Area under curve (AUC) of postprandial glucose responses of normal and streptozotocin-induced diabetic mice.

	AUC (mmol min/L)	
Group	Normal mice	Diabetic mice
2,7″-Phloroglucinol-6,6′-bieckol	828.78 ± 85.30^b^	2349.31 ± 129.17^b^
Acarbose	781.53 ± 109.34^b^	2231.67 ± 92.10^b^
Control	1038.53 ± 91.75^a^	2690.83 ± 122.88^a^

2,7”-Phloroglucinol-6,6′-bieckol (10 mg/kg), acarbose (10 mg/kg), and control (distilled water) were orally co-administered starch (2 g/kg). Each value is expressed as the mean ± S.D. of seven mice (*n* = 42).

^a,b^Values with different letters are significantly different at *p* < 0.05 in Duncan’s multiple range tests.

## Discussion

Elevated postprandial hyperglycaemia is caused by consumption of high-carbohydrate diets, which can progress to full symptomatic type 2 diabetes. One therapeutic method to decrease postprandial hyperglycaemia is reduction of absorption of glucose through inhibition of carbohydrate-digesting enzymes such as α-glucosidase and α-amylase in the digestive organs (Abesundara et al. 2004; You et al. 2004). α-Glucosidases hydrolyze disaccharides to monosaccharides while α-amylase hydrolyzes alpha-1,4-glycocidic bonds and splits up starch components such as amylose and amylopectin into smaller oligosaccharides and disaccharides (Casirola & Ferraris 2006; Kwon et al. 2008). In this regard, α-glucosidases and α-amylase play critical roles in carbohydrate digestion and absorption. Therefore, inhibition of α-glucosidases provides an effective antidiabetic option by targeting postprandial hyperglycaemia.

In the present study, the effects of 2,7″-phloroglucinol-6,6′-bieckol on activities of α-glucosidases and α-amylase were evaluated in order to investigate the possible use of 2,7″-phloroglucinol-6,6′-bieckol as an anti-hyperglycaemic agent. 2,7″-Phloroglucinol-6,6′-bieckol showed higher inhibitory activities against both enzymes compared to acarbose, with IC_50_ values of 23.35 and 130.04 μM against α-glucosidases and 6.94 and 165.12 μM against α-amylase, respectively. The strong inhibitory effect of 2,7″-phloroglucinol-6,6′-bieckol may be due to reactions with the enzyme. 2,7″-Phloroglucinol-6,6′-bieckol, which is a kind of phlorotannin, is a polyphenolic compound isolated from *Ecklonia cava*. Polyphenolic compounds such as phlorotannins are known to bind to various proteins to form complexes. Studies have reported that the hydroxyl groups in polyphenolic compounds could play important roles in inhibiting enzyme activities (Stern et al. 1996; Piparo et al. 2008; Xiao et al. 2013). Thus, the hydroxyl group in 2,7″-phloroglucinol-6,6′-bieckol may bind to enzyme active sites and inhibit enzyme activities.

Postprandial hyperglycaemia contributes not only to diabetes but is also an independent contributing factor to diabetic complications (Grundy et al. 1999). Based on the strong inhibitory results of 2,7″-phloroglucinol-6,6′-bieckol *in vitro*, we evaluated the effects of 2,7″-phloroglucinol-6,6′-bieckol on postprandial hyperglycaemia using STZ-induced diabetic mice. In our study, oral intake of 2,7″-phloroglucinol-6,6′-bieckol with starch significantly decreased postprandial blood glucose levels in STZ-induced diabetic and normal mice. Further, the 2,7″-phloroglucinol-6,6′-bieckol group showed a significantly reduced AUC in the postprandial glucose response compared to control diabetic mice. These results imply that 2,7″-phloroglucinol-6,6′-bieckol controls postprandial hyperglycaemia by delaying dietary carbohydrate absorption due to the inhibitory effects of 2,7″-phloroglucinol-6,6′-bieckol on carbohydrate enzymes.

Control of postprandial hyperglycaemia level is important not only in diabetic patients but also individuals with impaired glucose tolerance. Various epidemiological studies have suggested that postprandial hyperglycaemia might be more strongly correlated with cardiovascular morbidity and mortality than fasting hyperglycaemia (Bonora & Muggeo 2001; Chiasson et al. 2003). Therefore, alleviation of postprandial hyperglycaemia indeed plays an important role in controlling diabetes and preventing cardiovascular complications.

Acarbose decreases the requirement for insulin by controlling postprandial hyperglycaemia and can reduce the blood glucose level after meals. However, this hypoglycaemic agent has limitations and side effects such as flatulence, abdominal discomfort and diarrhoea (Clissold & Edwards 1988). Thus, there has been increased research into more efficacious agents presenting lesser side effects. Phlorotannin from marine algae become good source of natural anti-diabetic materials (Kim et al. 2008a). Several phlorotannins isolated from marine algae have the potential to prevent diabetes mellitus because of their high α-glucosidase and α-amylase inhibitory activities (Heo et al. 2009; Lee et al. 2014). These studies have shown similar results with our research that phlorotannin from brown algae may have a beneficial effect on controlling postprandial glucose levels. Therefore, this study suggest that 2,7″-phloroglucinol-6,6′-bieckol might be useful as a natural compound for treating postprandial hyperglycaemia.

## Conclusions

In summary, 2,7″-phloroglucinol-6,6′-bieckol showed strong inhibitory effects against α-glucosidase and α-amylase activities. Furthermore, 2,7″-phloroglucinol-6,6′-bieckol delayed digestion of carbohydrates and absorption in the small intestine, resulting in alleviation of the postprandial blood glucose level. Thus, 2,7″-phloroglucinol-6,6′-bieckol could be used as a potential nutraceutical for treating postprandial hyperglycaemia.
